# A Reinforcement Learning Model Equipped with Sensors for Generating Perception Patterns: Implementation of a Simulated Air Navigation System Using ADS-B (Automatic Dependent Surveillance-Broadcast) Technology

**DOI:** 10.3390/s17010188

**Published:** 2017-01-19

**Authors:** Santiago Álvarez de Toledo, Aurea Anguera, José M. Barreiro, Juan A. Lara, David Lizcano

**Affiliations:** 1 Escuela Técnica Superior de Ingenieros Informáticos, Campus de Montegancedo, Technical University of Madrid (UPM), Boadilla del Monte, 28660 Madrid, Spain; santiagoalvarezdetoledo@hotmail.com (S.A.d.T.); jmbarreiro@fi.upm.es (J.M.B.); 2 Escuela Técnica Superior de Ingeniería de Sistemas Informáticos, Technical University of Madrid (UPM), C/Alan Turing s/n (Ctra. de Valencia km. 7), 28031 Madrid, Spain; aanguera@eui.upm.es; 3 Escuela de Ciencias Técnicas e Ingeniería, Madrid Open University (MOU), Crta. de la Coruña km. 38.500, Vía de Servicio, 15, Collado Villalba, 28400 Madrid, Spain; david.lizcano@udima.es

**Keywords:** machine learning, reinforcement learning, ADS-B, perception-action-value association, air navigation

## Abstract

Over the last few decades, a number of reinforcement learning techniques have emerged, and different reinforcement learning-based applications have proliferated. However, such techniques tend to specialize in a particular field. This is an obstacle to their generalization and extrapolation to other areas. Besides, neither the reward-punishment (r-p) learning process nor the convergence of results is fast and efficient enough. To address these obstacles, this research proposes a general reinforcement learning model. This model is independent of input and output types and based on general bioinspired principles that help to speed up the learning process. The model is composed of a perception module based on sensors whose specific perceptions are mapped as perception patterns. In this manner, similar perceptions (even if perceived at different positions in the environment) are accounted for by the same perception pattern. Additionally, the model includes a procedure that statistically associates perception-action pattern pairs depending on the positive or negative results output by executing the respective action in response to a particular perception during the learning process. To do this, the model is fitted with a mechanism that reacts positively or negatively to particular sensory stimuli in order to rate results. The model is supplemented by an action module that can be configured depending on the maneuverability of each specific agent. The model has been applied in the air navigation domain, a field with strong safety restrictions, which led us to implement a simulated system equipped with the proposed model. Accordingly, the perception sensors were based on Automatic Dependent Surveillance-Broadcast (ADS-B) technology, which is described in this paper. The results were quite satisfactory, and it outperformed traditional methods existing in the literature with respect to learning reliability and efficiency.

## 1. Introduction

Artificial Intelligence (AI) is defined as any intelligence exhibited by man-made “mentifacts” or artifacts (until now, computers). One of the major goals of systems using AI is to somehow simulate human intelligence through knowledge acquisition. In order to do so, these systems need to perceive the information coming from their environment. Taking that into account, the use of sensors is absolutely necessary. The discipline of sensing has experienced an important growth in recent years, regarding sensor design, applications and technology.

Ever since they were first invented, intelligent systems were meant to acquire knowledge automatically. Machine learning is the branch of AI covering a series of techniques and concepts for building machines that are capable of learning. Machine learning has been one of goals most often pursued by AI in the past. According to Simon [1], a system is based on machine learning when, thanks to what it has learned, it is capable of executing the same task more efficiently next time round.

Reinforcement learning is an area of machine learning that draws inspiration from behavioral psychology. Reinforcement learning (also sometimes referred to as algedonic learning) studies how systems size up an environment in an attempt to maximize the notion of reward. This field then is based on the concept of reinforcement, which modifies a conditioned reflex or learned behavior by increasing (positive reinforcement or reward) or reducing (negative reinforcement or punishment) its occurrence.

Over the last few years, reinforcement learning has had a major impact as a great many techniques have been developed and applied in many different domains. It has not, however, been one hundred percent successful due to two major stumbling blocks in its development: (a) applications are specialized for a particular field and are thus hard to generalize and extrapolate to other areas; and (b) the learning process and convergence of results are slower and more limited than they should be. Sometimes learning itself generates so much information that the process breaks down.

In order to overcome the above difficulties, this research proposes a bioinspired model that is usable across different types of reinforcement learning applications irrespective of the input and output types used or their complexity. Additionally, it aims to blend a number of general principles that are useful for speeding up the above learning process.

To do this, our model is based on a central association module, an input module and an output module. The input module is responsible for sensing the environment and generating patterns based on the input stimuli that it feeds to the central module. These patterns are generated by sensors that set out to emulate perceptions by different living beings (optical, tactile, etc.) in nature. These sensors generate perceptions that are mapped to perception patterns. Similar perceptions gathered at different points of the environment under exploration are mapped into the same perception pattern. Decision-making complexity is reduced when similar perceptions are grouped within the same perception pattern. The central module feeds action patterns to the output module, which is responsible for performing the respective actions.

The central association module has mechanisms for statistically associating the input and output patterns that have occurred repeatedly and have provided positive or negative, right or wrong results, according to the bioinspired ideas proper to algedonic learning. To do this, it is fitted with results rating mechanisms, which react positively or negatively to particular sensory stimuli. The described association process, which will be formally defined later, drives the learning process. As the central association module is related to patterns of stimuli and actions rather than directly to particular sensory stimuli or specific actions, its features are generalizable enough for use in different applications and environments.

The proposed reinforcement learning model has been applied to the air navigation domain. Air navigation denotes all the techniques and procedures used to efficiently pilot an aircraft to its destination, without doing damage to crew, passengers or people on the ground. Because air navigation is a domain with strong limitations as far as experimentation and testing are concerned, we had to implement, at this first stage of our research, a simulated system including the proposed model. In particular, our system implements the sensors designed for the model by using a technology known as automatic dependent surveillance-broadcast (ADS-B), a surveillance technology described throughout this paper. In addition, our system has collision avoidance and automatic navigation capabilities, and has been tested on several real-world flight scenarios. The aim within these scenarios is for the aircraft to safely, effectively and automatically reach its destination avoiding collision with obstacles and other aircraft.

As we will see later, the results reveal a high success rate with respect to learning (and therefore the safety and reliability of the simulated air navigation system). It is more efficient than other techniques reported in the literature in terms of learning time and reliability after learning. It also has other benefits that are intrinsic to the method and will be outlined at the end of the paper. In our view, ADS-B technology, leading to the direct and efficient implementation of the sensors on which the proposed model is based, is a crucial factor in the achievement of such good results and an incentive for its future implementation in other fields.

The remainder of the paper is organized as follows. Section 2 presents key research and concepts related to the topic of this paper; Section 3 explains the proposed model; Section 4 describes the implementation of the simulated system (based on the model proposed) in the air navigation domain and the technology on which it relies: ADS-B; Section 5 illustrates the test scenarios and reports the results; Section 6 analyzes the applicability of the proposal, where it is compared with other existing options, describing its strengths and weaknesses; and, Section 7 outlines the conclusions and future research lines.

## 2. Related Work and Concepts

As already mentioned, this paper describes a reinforcement learning model based on a perception module that uses sensors. We also describe the implementation of a simulated system that uses the proposed model. The sensor technology used in that system is based on ADS-B.

ADS-B is a surveillance technology that is used to determine an aircraft’s position by means of satellite navigation, typically the global positioning system (GPS). This position is then broadcast at regular time intervals to inform other agents of the aircraft’s position [2].

In a traditional air navigation environment, these other agents are usually ground air traffic control stations and other airborne aircraft. Thanks to this technology, different air traffic operations can be managed from the ground as it reports the position of nearby aircraft. It also enables an aircraft to make decisions on flight path changes if there are other aircraft in its path.

**Figure 1:**
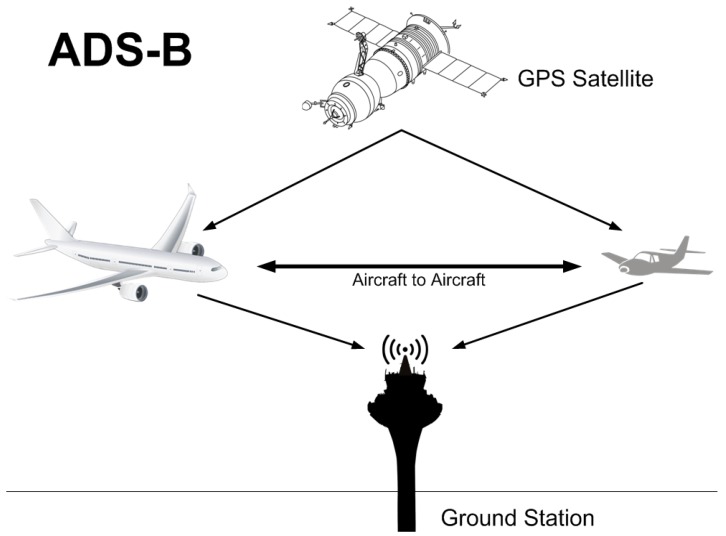
Standard ADS-B (Automatic Dependent Surveillance-Broadcast) operation.

Figure 1 illustrates this classical model described above.

As Figure 1 shows, ADS-B is based on the use of two types of airborne components: (i) a GPS source; and (ii) a data link (ADS-B unit) for information exchange.

As this technology is a mainstay of our paper, the technical details of the proposal and its application to the reference domain, air navigation, are described in Section 4.

With regard to the concepts related to the learning process of this proposal, note that the proposed model aims to solve problems with an active agent in an inaccessible environment, where environmental knowledge is incomplete. In this scenario, most techniques assume that there is no environment model (model-free reinforcement learning).

Table 1 describes prominent model-free reinforcement learning methods.

Michie and Chambers used Monte Carlo methods in their cart-pole application. They used the mean times of each episode (balanced pole) in order to check which action was useful in which state and thus control which action to select [10]. Other authors have used these methods [11], sometimes for a special purpose, for example to solve linear systems of equations [12]. A review of the literature published over the last ten years did not reveal many proposals by the reinforcement learning community related to this type of methods.

Over the years, different authors have proposed temporal difference-based techniques following the above idea [9,13]. Although not a paper specializing in temporal differences, the research reported in [14], proposing an online policy iteration algorithm that evaluates policies with the so-called least-squares temporal difference for Q-functions (LSTD-Q), does use concepts related to this approach.

As developed by Watkins, Q-learning combined the trial-and-error, optimal control, dynamic programming and temporal difference learning methods. However, Werbos had put forward a convergence between trial and error and dynamic programming earlier [15]. Action-value methods were first proposed for the one-armed bandit problem [16]. They are often referred to in the machine learning literature as estimation algorithms. However, it was not until later that Watkins introduced the method and the actual action-value term [17]. Watkins later conducted a thorough convergence test [9]. Other authors also tested several forms of system convergence [13]. The reinforcement learning community has proposed and improved a number of different methods based on Q-learning ideas over the last ten years [18,19,20,21].

The above three approaches have been used (in their original form or with slight modifications) successfully over the last few years. For example, reinforcement learning has been applied to the game of backgammon [22], elevator control [23], the field of autonomic computing [24], the game of Go [25], epilepsy treatment [26], and memory controller optimization [27].

## 3. Proposed Model

The reinforcement learning approaches described at the end of Section 2 are not generally effective enough for most applications of any complexity. General-purpose versions combining several such methods and tools are tailored to the respective application. Far from being a generally applicable approach, the application of reinforcement learning has come to be a practice of manually adapting different methodologies. Richard Sutton went as far as to say that there is often more art than engineering in having to tailor such a tightly fitting suit taking on board so much domain-specific knowledge [28]. Thus, there appears to be a need for a more general model for different application domains and areas.

Another area where there is room for improvement is the adoption of ideas with respect to action and state hierarchy and modularity. If we look at actions like tying a shoelace, placing a phone call or making a journey, for example, not all learning consists simply of one-step models of the dynamic environment or value functions. Although these are higher level scenarios and activities, people who have learned how to do them are able to select and plan their use as if they were primary actions. Similarly, we need to find a way of dealing with states and actions, irrespective of their level in a hierarchy and their modularity.

Additionally, there is still a rather evident need for methods that avoid an information explosion, somehow retaining only the key or vertebral parts of the experiences.

The model described in this paper was proposed with the aim of addressing the above limitations detected in the literature. Although we have not found any specialized literature on reinforcement learning dated later than 2012, a definitive solution to the problem addressed here, especially with regard to the need for broader applicability, has not yet been found (i.e., only specific improvements have been addressed). This is one of the key objectives of this proposal.

### 3.1. General Operation of the Proposed Model

**Figure 2:**
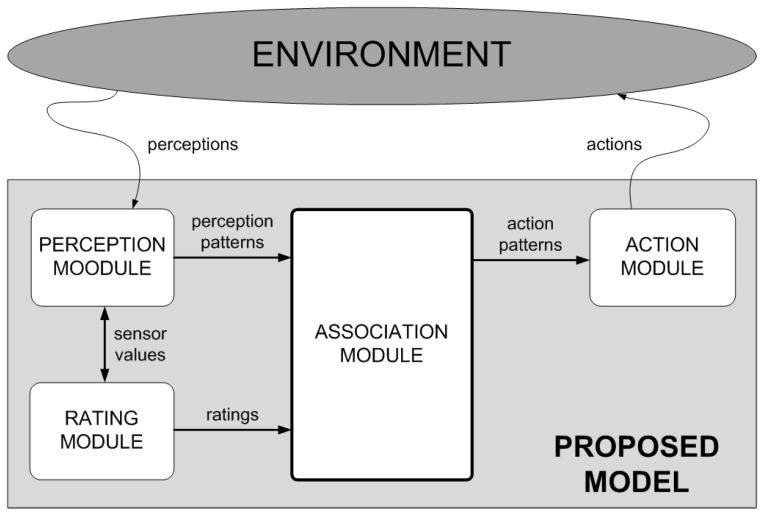
Overview of the proposed model.

As Figure 2 shows, the structure of the proposed model is clearly modular. On the one hand, the proposed model has two parts specializing in interaction with the environment: the perception and action modules.

The perception module is responsible for perceiving the environment through sensors and transforming each perception of each particular instant into a specified perception pattern. As we will see later, the model will manage this pattern to determine which action to take. In order to perceive the environment, the model provides for the use of sensors. These sensors aim to emulate the way in which human beings perceive their environment (by means of senses such as touch to detect when different surfaces come into contact, sight to detect distances to objects and other agents, hearing to detect possible movements, etc.). These sensors are set up based on the special requirements of each problem and the characteristics of the environment in question. Section 4.1 describes an application domain (air navigation), which illustrates, apart from the characteristics of the sensors, the importance of mapping different perceptions (with similar characteristics but gathered at different points of the environment) into the same perception pattern. As we will see in Section 4.1 through different examples, each perception pattern is composed of the different elements perceived by the sensors within the agent range, the relative position of those elements with respect to the agent (left/right) and whether the agent is further or closer to those elements in the current cycle compared to the previous one. Section 4.2 reports the technology on which these sensors rely.

On the other hand, the action module is responsible for executing the action selected once the perception pattern has been processed in the environment.

Although generally applicable, these perception and action modules can be parameterized in each domain to establish aspects like the range of perception, the maneuverability of the agents in the environment, etc.

The model also has a rating module. This module is responsible for establishing the reinforcement (reward or punishment) for the current situation in view of the action that has been executed and its effect on the environment. The parameters of this module should be set for each domain, although the user will have to set no more than the rating (sign and magnitude) of each object type identified by the agent in the environment.

Finally, the central part of the model, responsible for operating on the abstract knowledge is referred to as the association module. An association (normally denoted as a P-A-V association) is a value (V) that links each perception pattern (P) with each possible action that can be taken (A) in such a manner that this value is used to decide, for each pattern, which is the better action in the current scenario. The model uses a matrix composed of as many rows as perception patterns (P) and as many columns as possible actions to take (A). Each cell of this matrix stores a value (V) that represents the reward (positive or negative) of taking the action A in the presence of pattern P. As we will see later, this matrix is updated as the agent learns. In addition, this matrix is read by the action module in order to choose, in the future, the action A’ that returns a higher positive reward in the presence of pattern P. In other words, it selects the action A’ corresponding to the maximum value found in the matrix for row P. All this process will be more formally described in Section 3.2.

The last two modules are completely domain independent, although they can be configured to be able to establish the value of positive and negative reinforcements, etc.

**Figure 3:**
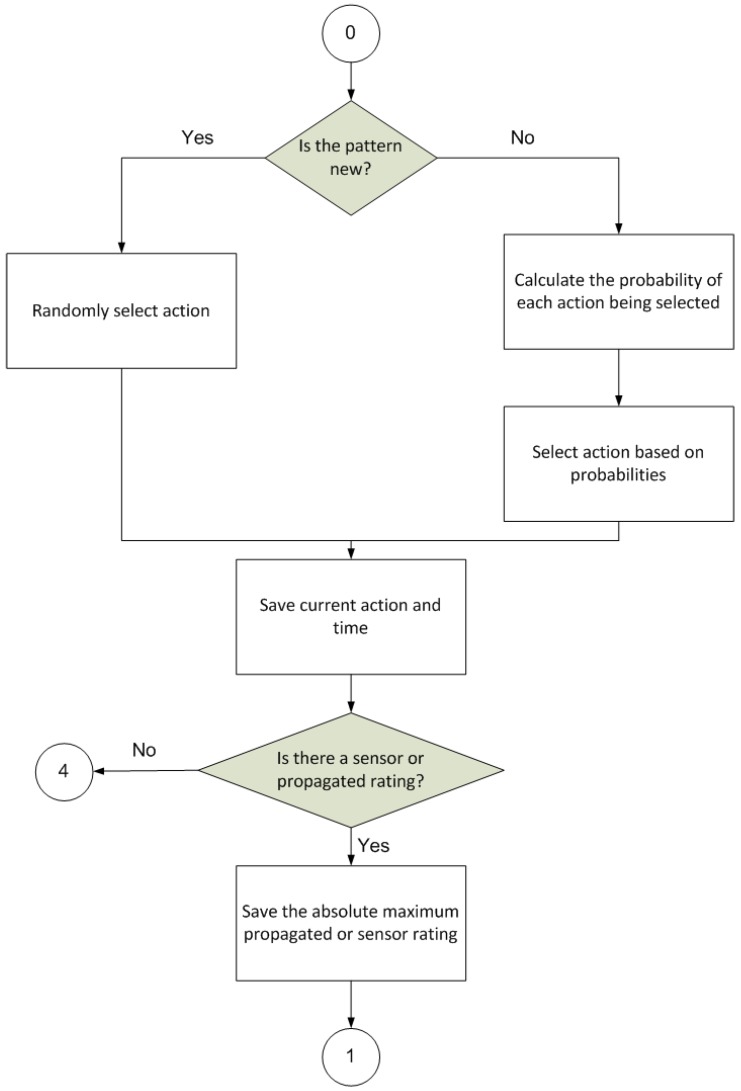
Action selection diagram (0 is the initial state of this diagram; state 1 continues in Figure 4; state 4 continues in Figure 6).

**Figure 4:**
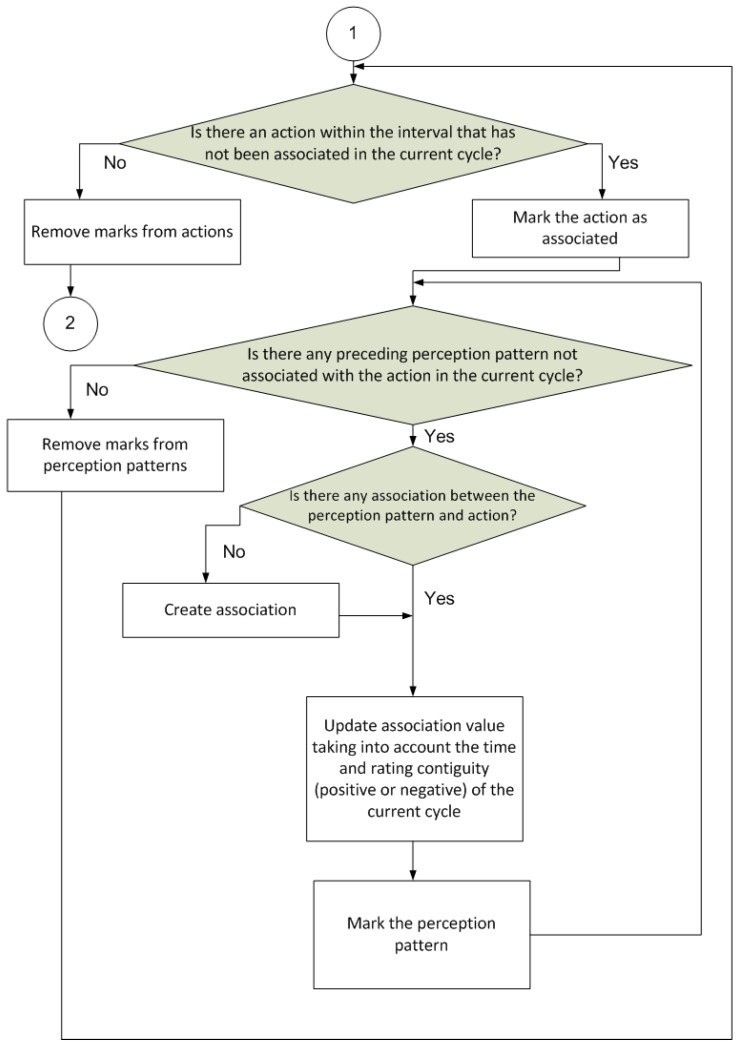
Association updating (1 is the initial state of this diagram; state 2 continues in Figure 5).

**Figure 5:**
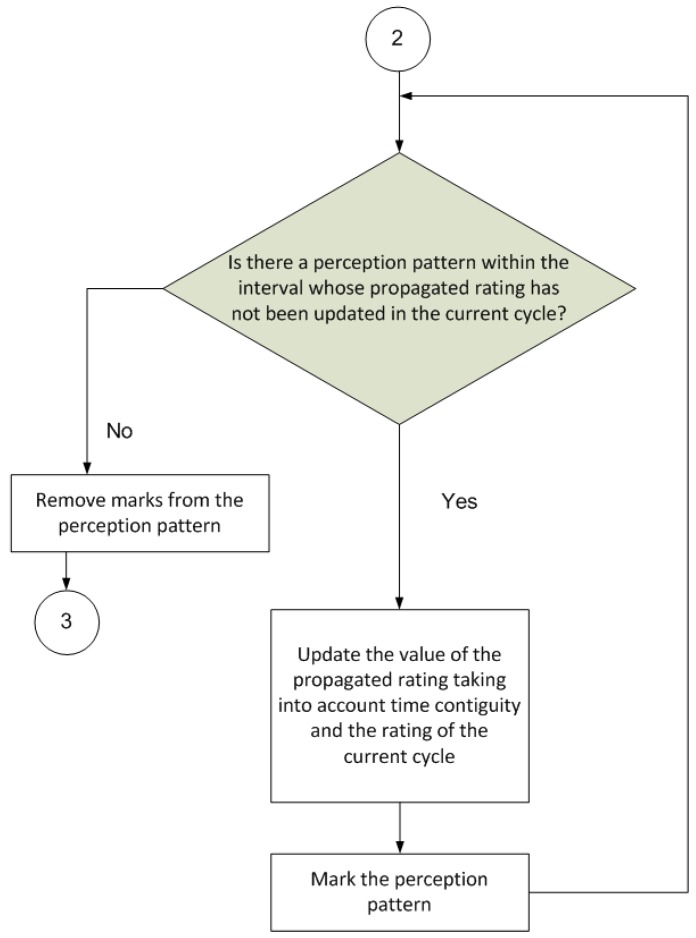
Rating back-propagation (2 is the initial state of this diagram; state 3 continues in Figure 6).

**Figure 6:**
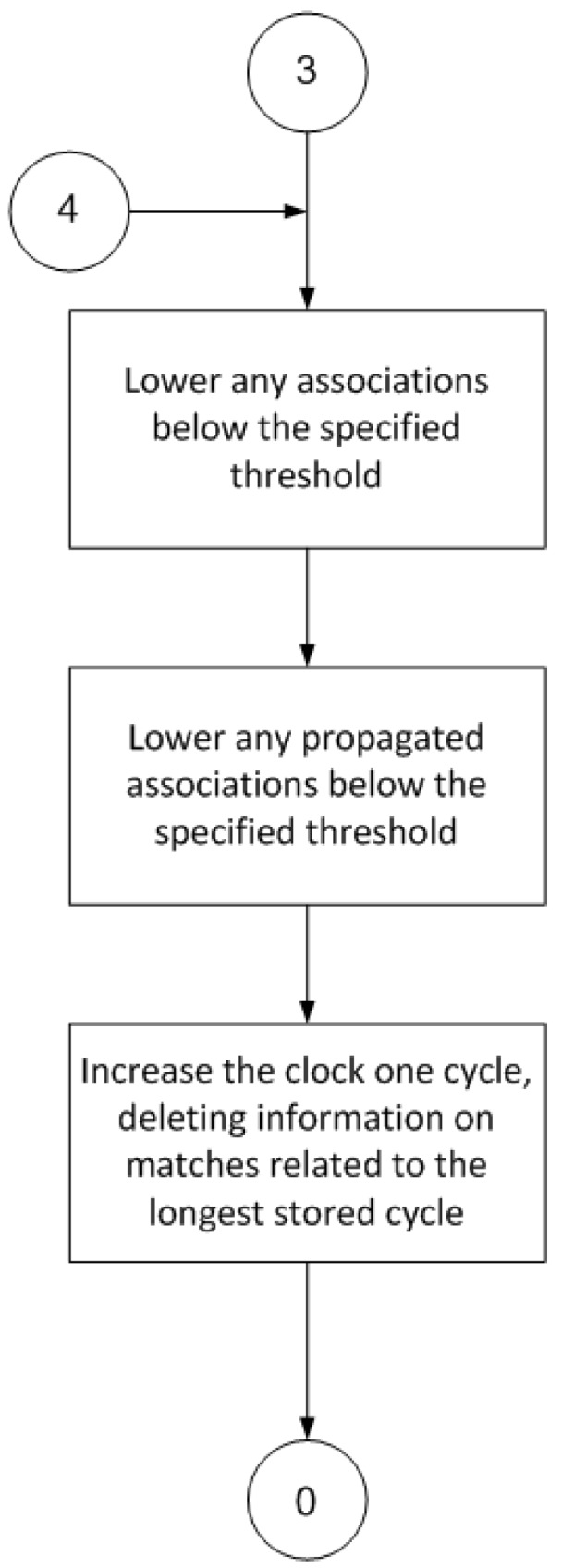
Deleted information (3 is the initial state of this diagram; state 4 comes from Figure 3; 0 is the final state of this diagram and it is the initial state of Figure 3 at the same time).

The operation of the proposed model is based on a procedure that is divided into cycles, where each cycle is composed of several separate steps (Figure 3, Figure 4, Figure 5 and Figure 6).

Figure 3 illustrates the first step of the procedure, whose final goal is to select an action for application within the environment.

It has to take a number of intermediate steps on the way to selecting this action. First, the stimulus is perceived and inspected to check whether it matches a known or constitutes a new input pattern. If it is new, the probability of the different actions available in the procedure being selected will be evenly distributed. If, on the other hand, the pattern is already known, the action that is considered best according to the knowledge accumulated by the procedure will have a greater probability of being chosen.

Figure 4 shows the next step of the procedure. This is to update the associations between the perception patterns and actions. As explained earlier, a specified value, which is referred to as the association, between each perception pattern and all the different actions is stored. This value is then used to decide which action should be selected, as described in the first part of the procedure. Each association is calculated based on the rating output after executing each action in response to a particular perception pattern (the stronger the positive rating, the more likely the action is to be selected).

The next part of the procedure (Figure 5) aims to propagate the rating perceived in the current cycle back to patterns perceived in the cycle immediately preceding the current cycle. Thus, the gathered knowledge is much more grounded; for example, if a good result is achieved after executing a series of actions, all preceding actions and not just the action immediately prior to the result are rated positively. The same would apply if the result of executing a series of actions were negative.

Finally, the last part of the procedure (Figure 6) deletes any superfluous information, namely, associations with a very low value that are of little use in later cycles, and propagated ratings that are below a specified threshold and are thus useless to the learning process.

These steps will be executed as often as necessary until the specified goal has been achieved (thereby solving the problem) or the maximum number of iterations is reached if the agent does not learn successfully.

### 3.2. Proposed Model Execution Cycle

Section 3.1 gave a preliminary and general description of how the procedure implementing the proposed model works. This section describes this procedure more formally.

The proposed model is based on a cyclical perception-action-value association process. This cycle will be executed until the final objective is achieved or the user-specified maximum number of iterations is reached. A maximum number of cycles is established for each trial. The trial will have failed if the objective is not achieved within this number of cycles. Even so, any learning is retained for the next trial. A maximum number of trials per simulation can also be set.

The steps executed in each cycle are explained in Algorithm 1.

**Algorithm 1.** Perception-action-value association process    **INPUT**: Environment, agent’s position in environment    **OUTPUT**: Selected action    **PRECONDITION**: Environment is accessible to the perception module    **POSTCONDITION**: The selected action is executed in the environment (step 2), the pattern associations are updated (step 4) and associations are selectively deleted (step 5).    **STEPS:****1**.**Perceive environment, call input pattern or patterns**The perception module sensors perceive the environment and generate the perception pattern associated with the state of the environment at the current time.**2**.**Select action**This step calculates the probability of each action being selected for a given input pattern. The probability of each action is weighted based on the values of the associations and/or reinforcements for the respective action and input pattern (P-A-V). If there is as yet no association and the rating is zero (neither positive nor negative, that is, there is no reinforcement), a default probability is assigned. If the rating is below a negative threshold, the assigned selection probability is 0. However, if all the possible actions are below the negative threshold, a probability of 1 is assigned to the least negative rating.**3**.**Calculate result rating**The perception pattern and action pattern pairs are associated based on the value of the result output during the action time cycle or later. The weight associated with this value decreases depending on the number of cycles in which the result occurred with respect to the perception and with respect to the action, according to Equation (1).(1)$V{\left(p_{,}a\right)}_{i}=V{\left(p_{,}a\right)}_{i-1}+\alpha ,$
where *V* is the value of the pair association between the perceived pattern (*p*) and the action taken (*a*) as determined by the rating system, *i* is the cycle in which the value occurs and *α* is the discount value per cycle that is applied to align the resulting value with the historical value. Variable *α* is parameterizable and specified by the user. Clearly, the action taken immediately before the perceived pattern (cycle *i*) will be rated higher than the preceding action (cycle *i* − 1). To be precise, it will be *α* units higher. Therefore, the action taken immediately before perceiving a pattern will be rated higher than the preceding actions. This suggests that the action taken in response to a perceived pattern is important, as are, albeit to a lesser extent, the actions leading up to that situation.**4**.**Update/generate P-A-V association weight**Each perception pattern has a unique value that depends on the value of the result perceived later, its magnitude and sign, and the number of cycles between the result and the perception. At the same time, the higher the certainty of the value being associated with patterns is, the larger the part of the value back-propagated towards preceding perception patterns will be. This is denoted by Equation (2).(2)$V_{P_{i}}=\frac{c_{p_{i-1}}V_{P_{i}-1}+\frac{\alpha }{2^{j}}}{c_{p_{i-1}}+\frac{\alpha }{2^{j}}},$
where *V_Pi_* is the value of the perception pattern in the current cycle, *α* is the discounted value depending on the elapsed *j* cycles, *c_Pi−1_* is the certainty of the perception pattern value in the preceding cycle, *V_Pi−1_* is the value of the perception pattern in the preceding cycle. Parameter *α* appears again in this equation and is used for the same purpose as in Equation (1). In this case, fraction *α/*2^j^ will be higher for a smaller number of elapsed cycles, *j*, between the perceived pattern and the preceding actions, that is, the association will be stronger the closer the pattern and the action are in time. The above certainty of such a value for the perception pattern depends on the actual value of the result and the number of cycles between the result and the perception (measurement of time). This is denoted by Equation (3).
(3)$$c_{p_{i}}=c_{p_{i}-1}+\frac{\alpha }{2^{j}},$$
where *c_pi_* is the certainty and, again, *α* is the discounted value depending on the elapsed *j* cycles.**5**.**Delete associations**Associations are deleted by periodically lowering the weight level of the associations. This ends up deleting any associations that have occurred less often and pinpointing associations that are more common and/or stronger rated and/or have shorter time lapses between their parts.

As mentioned above, one of the key problems of reinforcement learning applications is how to deal with the combinatorial explosion when there are a lot of states and actions. To do this, the proposed solution selectively generates P-A-V associations attaching priority to any associations whose result ratings are stronger, irrespective of whether they are positive or negative. Thus, perception and action patterns whose results were not rated or were rated neutrally take least priority and may not even be associated. Subsequently, associations with stronger ratings are more likely to be generated. It is not just a question of the associations being weighted higher or lower on the strength of their rating, which is indeed the case and worth consideration; the crux of the matter is that associations may not even be generated. This is very helpful for reducing the volume of information that has to be handled. Additionally, the selection procedure attaches priority to the associations that are most necessary for intelligent action.

On the other hand, there is a periodic deletion mechanism, which gradually removes the lowest weighted P-A-V associations. In the first place, any associations that are the product of chance, association perceptions, actions and results that are not really related to each other will end up being deleted as they are not repeated. Likewise, low weights will assure that the least necessary associations will also end up being deleted, not only because they are not repeated but also because their results are weak. Finally, any associations with a lower certainty level may also end up being deleted because there is not much similarity between the perceived signals and the called pattern. The entire deletion process is therefore priority based and selective.

## 4. Model Deployment

### 4.1. Air Navigation: Current Application

Air navigation is a discipline whose main objective is the safe transportation of goods and passengers on aircraft. It is an especially challenging domain because of the required safety level. It is also very motivating because of public concern surrounding episodes caused by aircraft accidents, for instance.

On this ground, we have selected the air navigation domain as a benchmark for testing our learning model. For obvious reasons, any technological advance in air navigation has to be tested in a simulated environment first.

We built the proposed model by implementing a simulated air navigation environment in which different agents (aircraft) have to safely reach their target (namely, land at their destination).

The simulated environment is composed of a three-dimensional space composed of a grid across which the agents move. The environment is bounded by fixed planes: lateral, top (representing the maximum altitude at which it is safe to fly) and bottom (representing the ground).

As already mentioned, the objective of the agents is to reach their destination (generally, the head of the runway). A virtual approach cone is added at the head of the runway. To do this, we implemented a perception module that informs each agent of the distance to the target and alerts it to other aircraft or obstacles in its proximity.

Specifically, this perception module was equipped with the following sensors:

**● Radar:**

**Figure 7:**
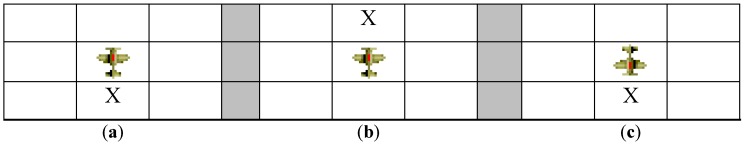
Example of three scenarios with obstacles.

This sensor provides information on any possible obstacle in the environment (ground, approach cone, and other agents) that is in the proximity of the agent within a specified maximum distance (radar range). Additionally, it reports negative ratings if such obstacles are too close to the agent, that is, are within touching distance, that is, the sign of the generated rating will be negative (a value less than 0). Radar operates as follows: the agent must do a full-circle scan in each cycle, taking note of all the obstacles that are positioned in its vicinity. The perception pattern is formed depending on the agent’s orientation with respect to each detected obstacle. It is important to ascertain whether there is an obstacle in its proximity and, if so, what its position is with respect to the agent, as shown in Figure 7. Figure 7 illustrates three scenarios, each with an agent (represented by the figure of an airplane) and an obstacle (represented by the symbol X). These scenarios denote the importance of orientation in radar perception. Thus, Figure 7a,c should have a different perception pattern, whereas Figure 7b,c should have the same pattern (obstacle ahead).

**● Sensor at Head of Runway:**

This sensor informs the agent about its orientation with respect to the landing runway. In this case, the perception is obtained by calculating the angle formed by the orientation vector linking the agent with the head of the runway. If this angle is 0°, it will mean that the agent is oriented straight towards the runway. This angle is calculated to determine the position of the runway with respect to the agent—(a) ahead; (b) to the right; (c) to the left; or (d) behind—where it is this discrete value that actually forms the pattern.

This sensor also provides information on whether the information is moving towards (positive rating) or away (negative rating) from the target. Although the simulation used the difference between distances to the target in consecutive cycles, this can be implemented in the real world using any kind of geopositioning system.

**Figure 8:**
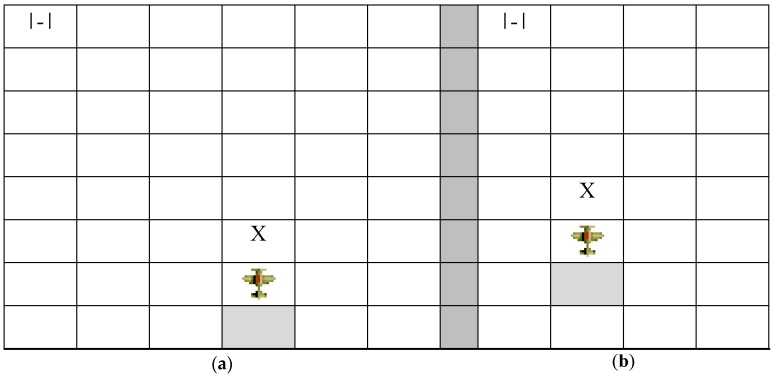
Example of two scenarios with obstacles and head of runway (the agent is closer to the runway in (**b**) than in (**a**) but the perception pattern is the same in both cases).

Figure 8 shows two pertinent scenarios involving the two sensors described above. The landing runway is represented in Figure 8 by means of the symbol |-|. In both scenarios, the agents meet with an obstacle head on as they move towards the runway (moving upwards in the diagram from the shaded box at their tail-end). Besides, as a result of the movement that brought the aircraft closer the obstacle, they managed to approach the head of the runway, which, in both cases, is to their left. The most important thing about these two scenarios is that both agents are actually perceiving the same perception pattern, even though the perceived realities are different (the agent is closer to the runway in Figure 8b than in Figure 8a, for example). This pattern is composed of the following information: *<Obstacle straight ahead, Runway to the left, Runway approach>.*

This approach, adopted in the proposed model, means that what are in actual fact different perceptions are regarded as the same thing, that is, the same perception pattern, if the salient features of the environment are perceived as being similar in both cases. This increases the efficiency and effectiveness of the learning process.

Thus, if the agent learns that the ideal maneuver in such a scenario is *<Turn left, Move forward>*, for example, it will tend to repeat this sequence of movements whenever it encounters this perception pattern, as it will be able in this manner to avoid the obstacle and move towards and approach the runway.

The above sensors could be classed as tactile and optical. The ADS-B technology on which they rely will be described in Section 4.2.

On the other hand, each agent is fitted with an action module composed of the following actions:

- Continue straight ahead: The agent moves to the next position in the direction that it is currently facing. The agent has a front and a rear, and moves in the direction that it is facing.
- Turn left: The agent stays in the same physical position as in the preceding cycle but turns (as it has a back and front) to its left a parameterizable number of degrees (a value of 15° was used in the test scenarios described later as it returned the best results).
- Turn right: As above, except that the agent turns the set number of degrees to the right.
- Ascend: As above, except that the agent turns the set number of degrees upwards.
- Descend: As above, except that the agent turns the set number of degrees downwards.

Each agent also has a rating module as described in the model. Approaching or steering towards the destination results in a positive reinforcement, whereas approaching obstacles, other aircraft or fixed planes results in a negative reinforcement. In both cases, reinforcement parameters are adjustable.

The association module is fully generic, as described in the proposed model.

It has all been implemented using the C++ programming language and different versions of the Visual Studio development environment. The system includes two basic packages: one for simulation and the other for visualization.

The simulation package offers a user interface for environment configuration: initial position and destination of the aircraft, possible obstacles (location and size), turning angle, reinforcements, etc. In order to evaluate the learning rate, the user can select the number of times (trials) that each simulation will be repeated (the agent does not know anything at all about the environment at first and does better in successive trials, as it learns). Each trial is composed of a maximum number of cycles, during each of which the procedure described in Section 3.2 will be executed. After each cycle, the agent will perform the maneuver denoted by the selected action, where there is a one-to-one correspondence between the cycle and maneuver. If the agent manages to achieve its objective before reaching the established maximum number of maneuvers, the trial is said to have been successfully completed. Otherwise, it is a failure.

**Figure 9:**
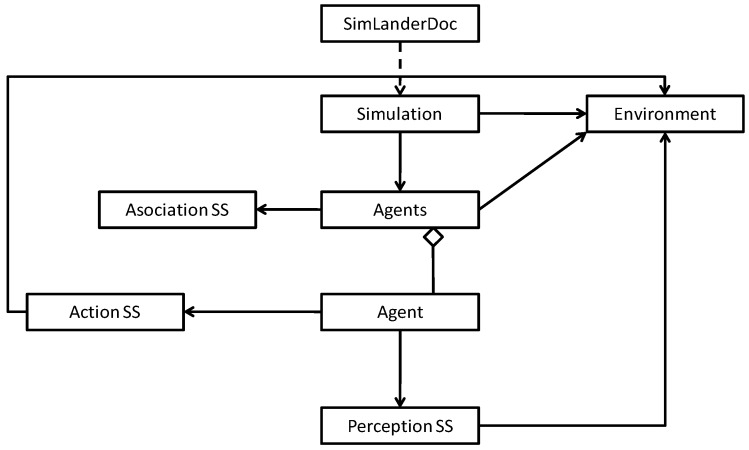
Simulation package class diagram.

For example, Figure 9 shows a snippet of the class diagram for the simulation package containing the most important classes (agents, model modules, environment and simulation, etc.).

As the simulation is executed, the system stores the simulation data in a binary file. Basically, this file stores the position and movement of the agent for each cycle of each trial. This procedure is enacted until the simulation ends when the user-defined number of trials is reached.

**Figure 10:**
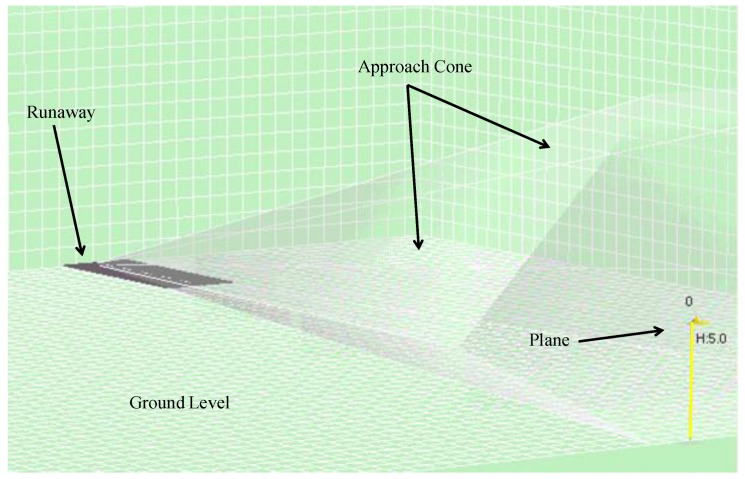
Simulation visualization tool.

The visualization system, on the other hand, is capable of replaying the simulation from the file in a visual environment that recreates the simulation environment (Figure 10). Users can browse the different trials and execute the simulation step by step to observe the movements of each agent in the environment. Figure 10 shows the agent (on the right), the different fixed planes (illustrated by the grids), the head of the runway (on the left) and the approach cone (which is really a hexagonal pyramid with a vertex on the head of the runway).

**Figure 11:**
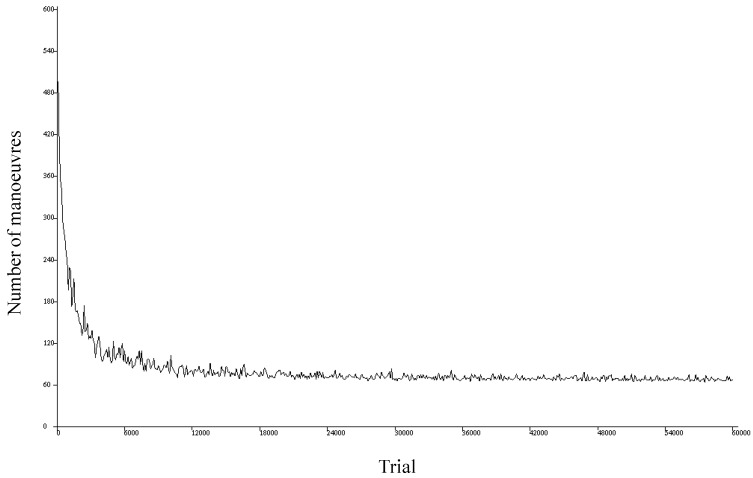
Example of plot associated with a simulation.

The visualization system also generates plots showing the number of maneuvers performed by each agent in each trial. Figure 11 shows an example of this type of plot, where the horizontal axis represents the different simulated trials and the vertical axis denotes the number of maneuvers performed in each trial. This type of representation is helpful for identifying the successes and failures of the simulation and checking the learning evolution.

Figure 11 shows the plot for a simulation of 60,000 trials where the agent has to achieve its objective within a maximum of 600 maneuvers for each trial. We find that the first trials end in failure (the objective is not achieved after performing the first 600 maneuvers). As the trials advance, however, the agent learns, and the number of maneuvers required to achieve the objective decreases until it stabilizes around 60.

### 4.2. Underlying Sensor Technology

The reinforcement learning model described here has been validated in an air navigation environment. In view of the features of this very special domain, we opted to implement a simulated environment, which is, in any case, a usual and necessary practice in such a sensitive area.

Simulation also has the advantage that the proposal will not become technologically obsolete, whereas a real environment requires the use of technology, which, according to Moore’s law (applicable primarily to computers but also extendible to other technological fields with minor changes), has an expiry date.

It is also true, however, that the simulated environment is being transferred to the real world, starting with drones. They are being used as a test bed, with the ultimate aim of the adoption of the model in real aircraft. In both cases, the technology used will be ADS-B as mentioned above.

After studying different technological approaches, we chose this option because it has many advantages for this particular project and more general research. In this case, we want to be able to implement the sensors described with innovative technology that has institutional support and is destined to become the standard air surveillance technology in the coming years: ADS-B.

From a more technical viewpoint, the sensors considered in our proposal are used for any agent (aircraft) to be able to find out the position of other obstacles of interest:

- Other airplanes;
- Destination (landing runway); and
- Different obstacles (towers, cables, limited access zones, etc.).

This technology was chosen in preference to others because any aircraft can gather information about the position of the different types of obstacles listed above if they are equipped with a position-emitting system with ADS-B technology. This is all the information required for the aircraft learning model to work.

**Figure 12:**
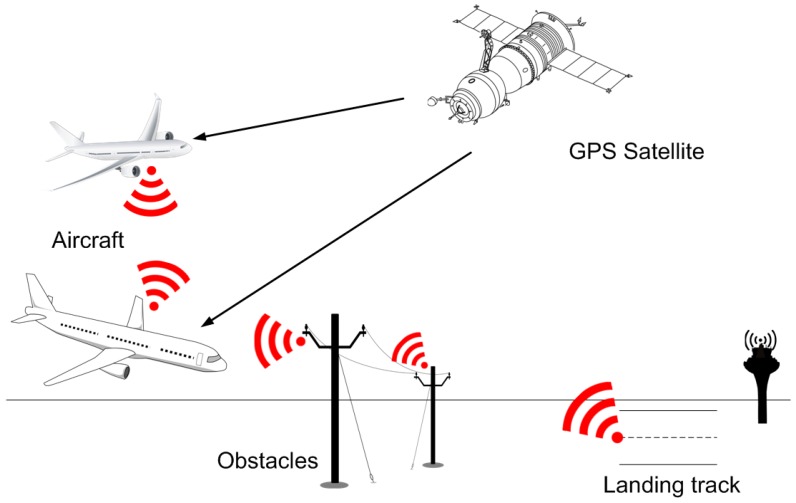
Adaptation of the original ADS-B (Automatic Dependent Surveillance-Broadcast) diagram to the designed proposal.

The most logical thing would apparently be to equip airports or landing runways, as well as, of course, aircraft with an ADS-B system. As regards objects that have the potential to block an agent’s path, it makes sense that they should also be equipped with such systems (they will really only need to broadcast their position), especially in an age where it is increasingly frequent for objects to be interconnected and intercommunicated (see the Internet of Things paradigm). All this would lead to a scenario, as shown in Figure 12, generalizing the conventional scenario illustrated in Figure 1.

We now describe the selected technology in detail. ADS-B is mainly based on the frequent and regular transmission of reports by means a radiobroadcast data link. ADS-B reports are periodically sent by the aircraft without any intervention whatsoever from the ground station. These reports can be received and processed by any receptor in the aircraft’s vicinity. In the case of the ground data acquisition unit, the ADS-B report will be processed together with other surveillance data and will be used for control operations.

ADS-B provides the option of sending the air-air or air-ground surveillance information. Direct air-air transmission means that a ground station does not have to intervene in order to carry out on-board aircraft surveillance tasks. Additionally, the use of ADS-B reports from other aircraft in the vicinity provides a clear picture of the air traffic status in the cockpit. ADS-B-transmitted surveillance data include the flight identifier, position, time and speed, etc.

There is no acknowledgement of receipt of ADS-B reports. Therefore, the aircraft does not know which receptors, if any, have received and are processing their reports. In fact, any aircraft or ground team in the vicinity may have received and be processing the information.

There are at present different ways of deploying ADS-B (they are all now being tested by EUROCONTROL), albeit with different levels of standardization and validation. The most widespread option is to use a Mode S or 1090 extended squitter transponder, as suggested by the International Civil Aviation Organization (ICAO) [29]. This is an extension of the traditional Mode S secondary surveillance radar, commonly used in other systems like the airborne collision avoidance system (ACAS). Accordingly, the aircraft regularly transmits “extended squitter” messages containing information like position or identification. The extended squitters are transmitted at the 1090 MHz secondary response frequency and may be received by any suitably equipped aircraft or ground station.

An ADS-B message currently has a length of 112 bits, as shown below.

1000110101001000010000001101011000100000001011001100001101110001110000110010110011100000010101110110000010011000

Each of the above bits has a meaning defined in Table 2.

The DF field identifies the message type. The above value should be 10,001 in binary (17 in decimal) for an ADS-B message.

DATA is another important field, which transmits the following information:

- Aircraft status: On ground or in flight (the number of squitters can be reduced if the aircraft is taxiing in the aerodrome, with the resulting reduction in frequency saturation).
- Position and speed (twice per second).
- Identification message, which should be constant (every five seconds if it is moving and every 10 s if it is stationary).
- Incident messages if necessary.

As regards system performance, its range is established at from 60 to 100 NM.

**Figure 13:**
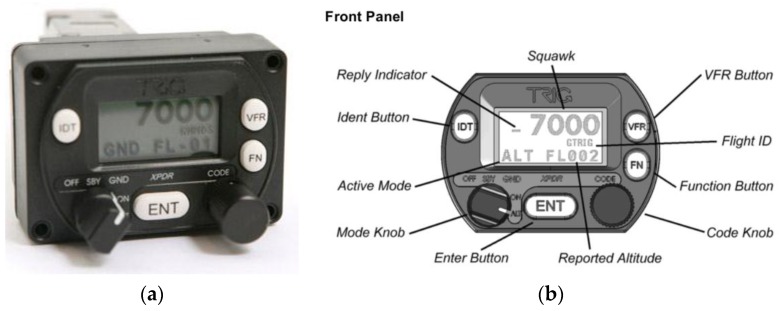
(**a**) Photograph of TT21 transponder; and (**b**) front panel of TT21 transponder.

In view of the above, we are using this technology, specifically Trig Avionics (Edinburgh, Scotland) Model TT21 Mode S transponders, similar to the one shown in Figure 13a, in our preliminary fieldwork. The transponder has the interface and controls described in Figure 13b.

The TT21 is an ideal transponder for experiments conducted on light aircraft, as it weighs around 450 g Additionally, it is reasonably priced (around €2000), making it viable for use in research.

The device specifications used are shown in Table 3.

**Figure 14:**
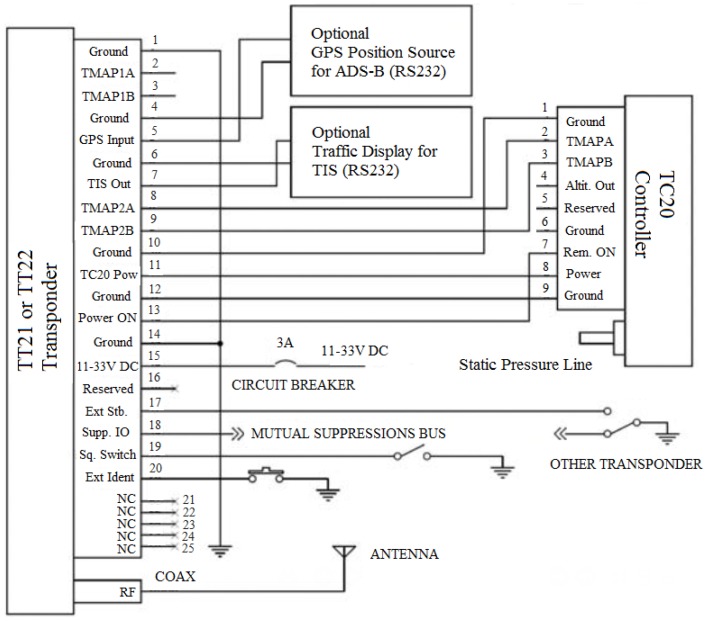
TT21 transponder connections.

As specified in Table 3, the transponder comes with a controller. In this case, the controller is connected directly to the reinforcement learning system proposed in this paper. Figure 14 shows the standard connections between the transponder and the controller. Figure 14 also shows that the transponder should receive the GPS signal through connection 5 in order to ascertain its position.

Although it is widely used and recognized, one of the key challenges faced by the Mode S extended squitter is that the transmissions may be confused with other Mode S functions, like elementary or improved surveillance or with ACAS, which also operate according to the same protocols and message formats and at the same frequencies (1030 MHz for queries and 1090 MHz for responses).

To conclude this section, we should note that, thanks to the joint efforts of different European (Single European Sky ATM Research-SESAR) and North American (Next Generation Air Transportation System-NextGen) bodies and other international institutions (like ICAO), the standardization levels of ADS-B technology are improving. In particular, its use in some areas of Australia is compulsory. There are plans in the United States for ADS-B adoption in some aircraft by 2020. In Europe (and particularly Spain), some airplanes will have to use it as of 2017. Canada has already adopted ADS-B technology for air traffic control. In the coming years, the evolution of this technology will determine the future of air navigation, as well as research, which, like the investigation reported in this paper, is reliant on its development and full standardization.

## 5. Experimentation and Results

### 5.1. Test Scenarios

Many simulations have been run on the implemented simulated environment recreating scenarios similar to what a pilot may encounter in his or her professional life.

In an air navigation environment, airline pilots have to safely pilot aircraft from a point of departure to a destination. To do this, they have to avoid any obstacles that they may encounter (other aircraft, buildings, mountains, storms, prohibited airspaces, etc.), making sure that goods and people (crew, passengers and ground staff) are kept safe and come to no harm.

We carried out different simulations in realistic piloting scenarios in order to test the proposed model. In this case, it is the intelligent agents implementing our model rather than the pilots that have to learn to choose the right path for the aircraft that they are piloting. Each simulation recreates a different scenario including diverse elements. These scenarios alternated the number of aircraft, the existence or otherwise of obstacles within the planned flight path, obstacle features, etc.

Overall, we carried out hundreds of simulations, of which nine representative examples are reported in this paper. Table 4 lists the name of the simulation, the number of participating agents, the number of obstacles in the planned flight path, and some observations to clarify the designed scenario.

**Figure 15:**
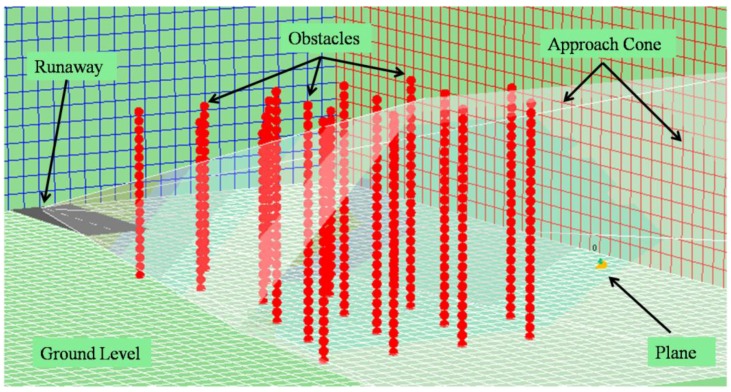
Visualization of the scenario designed in simulation Sim5.

**Figure 16:**
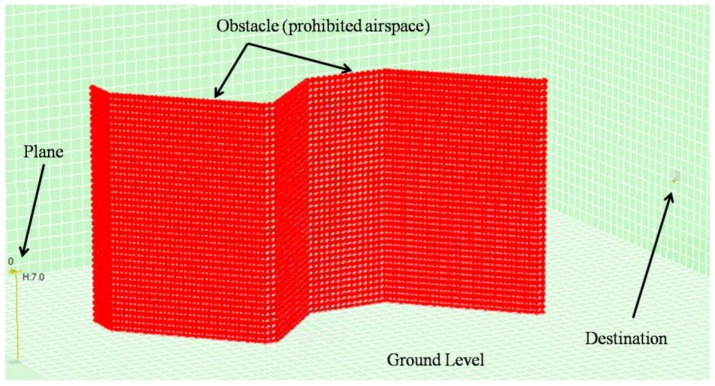
Visualization of the scenario designed in simulation Sim7.

In each of the simulations, 60,000 different trials were carried out. Due to the sheer volume of the generated data, one out of every 50 trials was sampled (60,000/50 = 1200 sampled trials). Each sampled trial was composed of 600 learning cycles in order to reach the target specified in the flight path of each aircraft. For example, Figure 15 and Figure 16 recreate the scenarios for simulations Sim5 and Sim7.

### 5.2. Results and Discussion

The benchmark used to evaluate the quality of the learning achieved by agents that adopt our model in the simulated environment included several quality indicators:

(a) Agent learning. An agent is considered have learned if the trial ended successfully (the aircraft reached its destination safely) in at least 99% of the last 10,000 simulated trials.
(b) The overall success rate (number of successful trials/total number of trials).
(c) Number of learning trials for all the different agents. The different agents are considered to have learned by a specified cycle when the success rate for the 500 trials preceding that cycle is greater than or equal to 95% for all agents.
(d) Total simulation time. In order to express the above indicator in more universal terms, we considered the total simulation time elapsed (in minutes) before the agents learned on the benchmark computer.
(e) Average agent learning time, calculated by dividing the total simulation time by the number of agents in the simulation. This is a more practical indicator, as the proposed procedure can be built into each agent separately and distributed, in which case the real learning time in a real distributed environment is the mean time per agent.
(f) Success rate considering the trials performed after agent learning.

Table 5 shows the results for these five indicators in each of the nine simulations.

We find that the agents managed to learn during the simulation and reached their target in all the simulations. To check the statistical behavior of the different quantitative indicators, we calculated the mean, standard deviation, and maximum and minimum considering all the simulations. The results are shown in Table 6.

Table 6 shows that there is a sizeable variance between learning trials and learning time, which makes a lot of sense in view of the disparity between scenarios and the clear relationship between their complexity and learning difficulty. However, when considering the time per agent (as we will see later in a more detailed study of this question), the results are less variable (dropping from 87.3 in overall terms to 34.8 when analyzed by agent). Additionally, the mean learning time per agent is perfectly feasible in computational terms: 26 min on average, after which the achieved learning is ready to be built into the real system for later use. As regards what is, in our view, the most important indicator (success rate after learning), it is never under 99%, with a mean performance of 99.7%. This confirms the reliability of the model in such complex scenarios such as the above. Furthermore, we find that, after learning, the model is positively stable and behaves rather well, as the deviation for the respective learning rate is low (no more than 0.3).

In the next part of the evaluation study, we compared our proposal with other state-of-the-art methods designed for similar environments described in Section 2: the Monte Carlo method (MC), temporal difference reinforcement learning (TD) and Q-learning (QL). The results of the comparison using the same quality indicators as above are shown in Table 7 (for all simulations, specifying the percentage value for the column headed Learned and the mean values for the other indicators).

As the above table shows, most of the compared methods (except MC) achieve 100% learning in the nine designed scenarios. The difference lies, however, in the time that it takes to achieve such learning and the behavior after learning. In this respect, our model is the one that learns on average in less time (26 min on average per agent versus 29.5 for the next fastest method). Besides, after learning, the proposed model is the one that generally exhibits a better behavior with a success rate of 99.7% compared with 99.4% for QL, which is the next best method.

**Figure 17:**
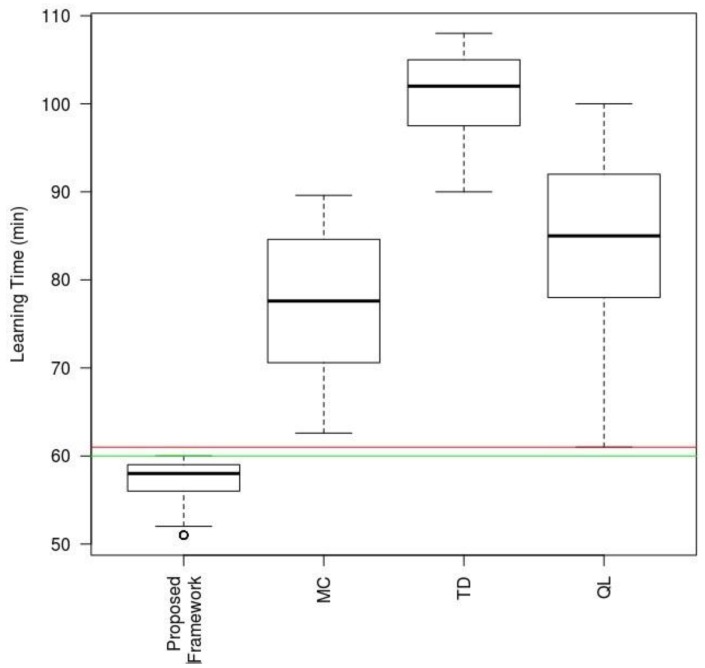
Boxplot comparison of results for Learning Time (minutes) variable (MC: Monte Carlo; TD: Temporal Difference; QL: Q-Learning).

In order to round out this study, we have built a comparative boxplot based on the analysis of the covariances of the study variables shown in Table 7 using only one explanatory variable: technique type used in the same scenario. The results are shown in Figure 17, illustrating the response variable: Learning Time taken by each of the techniques to achieve a 99% success rate after learning (threshold established in the ANCOVA model). The proposed model is the one that scores highest for the respective times, with a significance level α of 0.01%. We also found, from the resulting data, that, even in the worst scenario for the proposed mechanism, it still outperformed the best of the other techniques by several minutes.

**Figure 18:**
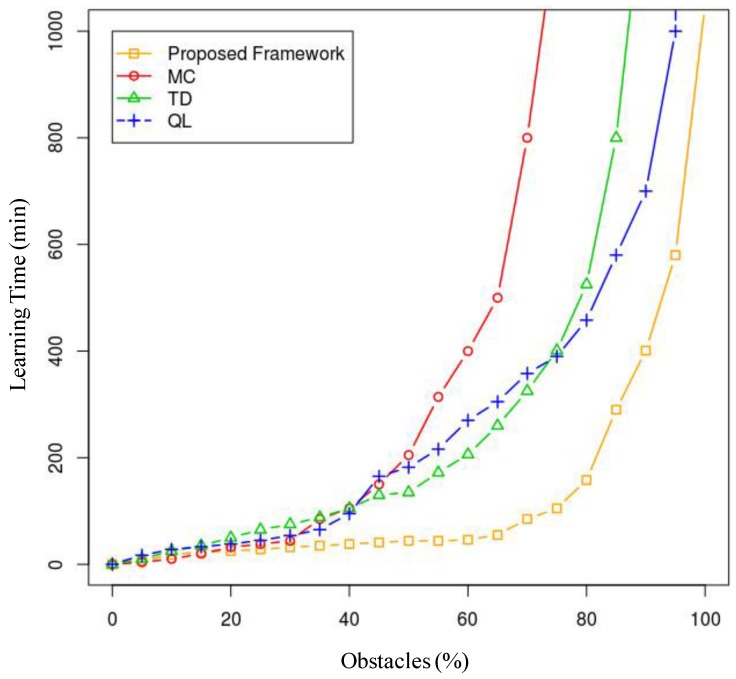
TT21 Comparison of the study results for the Learning Time (minutes) variable depending on the percentage of the environment that is occupied by obstacles (MC: Monte Carlo; TD: Temporal Difference; QL: Q-Learning).

Additionally, we conducted a study of each agent separately, calculating its learning time (time per agent) depending on the number of obstacles and the number of other agents present. In order to use a reduced dimensionality measurement that accounts for both scenarios, we calculated the learning time depending on the total percentage of occupied space in which the agent is to operate, where the 0% scenario is an obstacle-free scenario and the 100% scenario is a worst-case scenario. In this worst-case scenario, the agent will be unable to reach its destination because it is unable to maneuver in the environment. With this premise, we conducted an ANOVA study that analyses the variance of the results for each technique in more than 1000 simulated scenarios with each percentage point of occupancy, where these 1000 scenarios are random distributions of obstacles depending on the available space (a 500 × 500 two-dimensional grid). The results are shown in Figure 18.

As we can see, when the percentage of occupancy increases (over 85%), the learning times tend asymptotically towards infinity, as the space is sometimes completely blocked at some point. In any case, the technique that behaves better with regard to learning efficiency is the model proposed in the article, and this improvement is especially notable when the obstacles occupy from 35% to 80% of the available space. Over and above 80%, the model learning time results explode, and it is, in any case, inefficient. Under 35%, the results do not differ by many minutes, although, as demonstrated earlier, learning time will be lower in 99.9% of the cases using the described model.

The results for simulated scenarios that recreate real situations are satisfactory, especially as regards learning rate and efficiency, where it outperforms the classical state-of-the-art methods with which it has been compared.

In view of these results, the proposed model requires application in non-simulated air navigation environments that are closer to the real world.

## 6. Proposal Applicability

The reinforcement learning method proposed in this paper aims to improve upon some of the weaknesses of traditional reinforcement learning methods reported in the literature.

As explained above, our proposal uses associations (negative or positive numerical values) between each possible perception pattern and each possible action. The method selects the action to be taken in response to each pattern, attempting to maximize positive reinforcement. As shown by the experiments, our proposal outperforms other methods in terms of success rate and learning time. From the viewpoint of principles, our proposal also has a number of advantages over other methods.

The key difference between our proposal and Monte Carlo methods is that Monte Carlo methods require some previous experience of the environment. Additionally, unlike our proposal, Monte Carlo methods do not update associations step by step but take into account episodes. Moreover, the value of each state is the expected reward that can be gained from the respective state, whereas, in our case, the association is linked to the action for successfully exiting the respective state rather than to the actual state.

The ideas behind temporal difference methods are closer to the model proposed in this paper, as they also use the idea of updating ratings step by step. However, temporal differences are generally rated at state level. In our proposal, on the other hand, this rating is related not only to the state but also to the actions that can be taken to move to a yet more positive state.

Analyzing the above two approaches, our proposal is better suited to environments where there is no experience of the environment and where situations can occur suddenly (rather than for long episodes) and require a rapid response from the agent (step or action) in order to satisfactorily move to another, more positive state by executing the action. This applies, for example, to air navigation when an agent abruptly changes direction and threatens the safety of another agent or other agents.

Like Monte Carlo methods, our proposal also uses a ∈-greedy exploratory policy (the most promising action is selected, whereas a different action has a ∈ probability value of being selected).

The evolution of the above two approaches in the field of reinforcement learning led to the appearance of Q-learning. Instead of estimating how good or bad a state is, Q-learning estimates how good it is to take each action in each state. This is the same idea as considered in the proposed method. Q-learning methods work especially well in environments where there are a discrete number of states and a discrete number of actions. In continuous environments, however, where there are an infinite number of states, it is less efficient to bank on associations between states and actions and it may become unmanageable due to the huge amount of information. Our proposal, on the other hand, associates perception patterns with actions rather than states with actions. Note, therefore, that the perception system reported in this article discretizes the continuous environment into perception patterns. This leads to complexity reduction, as different states that have similar perceived elements are mapped to the same perception pattern.

Q-learning methods address the above problem differently. Instead, they use learning structures to determine which action to take in response to any state. Techniques based on deep learning, like deep neural networks, and other alternative approaches, like fuzzy inference systems, work particularly well in this respect. While we appreciate that deep neural networks are good at classifying and selecting which actions should be taken in each state, their structures are opaque, and the knowledge that they store cannot be specified. On the other hand, by maintaining a table of associations between patterns and actions, our proposal provides access to easily understandable knowledge that can be accessed at any time by the application domain experts. This works particularly well in critical domains like medicine or air navigation. In such domains, the agents concerned would not be at ease working with opaque systems like neural networks, as they would not be able to analyze the knowledge that led the system to take a wrong action (as required in critical environments). As regards fuzzy inference systems, we acknowledge the potential of soft computing techniques for improving the selection of actions for each perception pattern. In fact, as we will see in the future lines of research, it is one of the alternatives that are being weighed up for incorporation to the model.

Following on from the above, we briefly outline the circumstances under which the proposed model works particularly well below:

- There is no previous knowledge of the environment to be explored.
- The system has to respond to sudden circumstances occurring in the environment and needs to take on board the notion of short-term action in order to be able to deal with such circumstances positively.
- Actions should be or are more important (for moving to a better state) than the actual states in the environment.
- The number of states in continuous environments is infinite, and they need to be discretized as perception patterns.
- Opaque learning structures, such as neural networks, may be rejected by experts.

Indeed, the main contribution of this article with respect to the reinforcement learning area is the proposal of a method that, as the results (Section 5) and the above analysis of principles show, is recommended for use in the above situations where existing methods are insufficient or do not perform as well.

In other fields where no such circumstances occur, conventional approaches that, as reported in the literature, have proven to be applicable and useful, are applicable.

## 7. Conclusions and Future Lines

As explained throughout the paper, we propose a reinforcement learning model that perceives the environment by means of sensors which rely on ADS-B technology for application in the air navigation domain.

From the technological viewpoint, ADS-B is considered as the technology of the future in the field of air navigation surveillance. Its use brings research into line with navigation policies that will be adopted by most countries in the coming years, making this proposal more valuable and useful.

From the learning viewpoint, on the other hand, despite working well in their respective areas, many of the proposed reinforcement learning techniques are hard to adapt to deal with other issues for which they were not specifically designed. However, the model proposed in this paper has the big advantage that neither the module inputs nor the outputs are integral parts of the model. Notice also that thanks to input and output independency, more complex or general input and output modules can be coupled to basic input or output modules. Another noteworthy model feature is that it is unsupervised and adaptable to many different environments and applications. Finally, it is worth mentioning that the proposed model also quite successfully deals with one of the biggest problems of artificial intelligence methods: the combinatorial explosion of inputs and outputs

Although commercial air navigation would be too ambitious at this stage, we are planning to apply the proposed model using drones or RPAS (remotely piloted aircraft systems) in scenarios such as are described in this paper. In fact, we are now at the early stages of negotiations with sector companies and institutions with respect to the implementation of this proposal. All parties have shown a great deal of interest so far.

The use of ADS-B technology definitely facilitates the process of applying the proposed model in real environments as ADS-B resources are used to implement the sensors provided by the above model. However, ADS-B is still in its infancy, and it will take time for it to be adopted and consolidated as a standard technology. The research conducted over the coming years should allow for such a process of technology growth and maturity which may have a major impact on our proposal and its future applications.

As regards the proposed learning model, we are planning some research lines that could improve learning. The first is related to perception and perception pattern mapping (procedure step 1). This process is now performed mostly manually. We are considering using clustering techniques (data mining) to group similar perceptions with each other as part of the same pattern that could be represented by the cluster centroid or medoid. On the other hand, the associations are currently deleted (Step 5) when they are below a specified threshold. Generally, thresholds make problems binary (in this case, the association is retained or eliminated). This is not always flexible enough. As a line of this research, we plan to use more flexible techniques in this respect (fuzzy logic) in order to determine when an association should or should not be deleted. We intend to analyze the potential impact of using such soft computing techniques for association deletion on both the success rate and the computational efficiency of the learning process. Such techniques may also be useful in other parts of the proposed method (action selection, for example, as it is not always clear from the stored associations which action is to be taken). Accordingly, we also plan to deploy and evaluate the techniques in the above modules.

Finally, note that we have compared the proposed method with other existing methods at the level of results (Section 5.2) and concepts (Section 6) only. Therefore, a more formal and mathematical validation is required to be able to analyze the properties of the proposed method separately and compared with other existing methods. The project will focus on this line of research in the coming months.

## Figures and Tables

**Table 1:** Prominent model-free reinforcement learning methods.

Method	Description
Monte Carlo methods (Ulam and von Neumann—1940s) [3,4,5]	Monte Carlo methods are non-deterministic methods used to simulate complex problems that are hard to evaluate accurately. To do this, such deterministic problems are randomized using a random number generator. As far as we are concerned, Monte Carlo methods are a category of reinforcement learning approaches requiring experience—a sample of sequences of states, actions and results—instead of a full model of the environment dynamics.
Temporal difference (Sutton—1980s) [6,7,8]	Temporal difference methods perform reinforcement learning based on different successive predictions of the same value as time elapses. These methods perform what is known as bootstrapping. Even so, they differ from Monte Carlo methods in that they do not need to wait until the end of an episode and their terminal reward, but learn incrementally.
Q-learning (Watkins—1980s) [9]	Q-learning uses the value-action function instead of just the value function in order to predict the reward *Q* of a specified action in a specified state. Therefore, it takes into account the values of the action-state pairs rather than just of the states.

**Table 2:** ADS-B message structure.

Bits	Name	Abbreviation
1–5	Downlink Format	DF
6–8	Message Subtype	CA
9–32	ICAO aircraft address	ICAO24
33–88	Data frame	DATA
89–112	Parity check	PC

**Table 3:** TT21 technique specifications.

	TT21—Mode S for Light Aviation
Type	Transponder Class 2 Mode S level 2els ADS-B Class B0
Certification	ETSO C88A, C112C, C166A and TSO C88b, C112c, C166b, approved for IFR and VFR flight
Compliance	ED-73C, DO-160F, DO-178B Level B, DO-254 Level C, DO-260B, DO-181D
Supply voltage (DC)	9–33 V
Typical Consumption (at 14 V)	idle: 0.15 A active: 0.28 A
Nominal Transmitter Power	130 W at connector
Operating temperature	for the transponder −40 °C to +70 °C for the controller −25 °C to +70 °C
Cooling Requirement	no fan required
Weight	440 g
Dimensions	controller: H 44 × W 63 × L 54 mm transponder in tray: H 48 × W 68 × L 160 mm

**Table 4:** Description of the designed learning scenarios.

Simulation	#Agents	#Obstacles	Observations
Sim1	1	0	Basic simulation
Sim2	2	0	Both agents have to land on the same runway
Sim3	1	1	Narrow obstacle
Sim4	2	1	Narrow obstacle; both agents have to land on the same runway
Sim5	1	20	Narrow obstacles
Sim6	2	20	Narrow obstacles; both agents have to land on the same runway
Sim7	1	1	Large obstacle, emulating a prohibited airspace
Sim8	2	1	Large obstacle, emulating a prohibited airspace; both agents have to land on the same runway
Sim9	4	1	Medium-sized obstacle positioned mid-way along the flight paths of the four aircraft (with different overlapping flight paths)

**Table 5:** Results for the proposed model in each of the simulations.

Simulation	Learned	Overall Success Rate	Learning Trials	Learning Time (minutes)	Time per Agent (minutes)	Success Rate after Learning
Sim1	Yes	99.9	500	1	1	100
Sim2	Yes	99.7	4700	3	1.5	99.9
Sim3	Yes	99.7	3200	2	2	100
Sim4	Yes	99.2	9100	7	3.5	99.8
Sim5	Yes	99.4	8300	5	5	99.8
Sim6	Yes	98.9	12,450	12	6	99.5
Sim7	Yes	99.5	9400	74	74	99.7
Sim8	Yes	98.3	16,200	163	81.5	99.3
Sim9	Yes	98.1	14,850	239	59.8	99.1

**Table 6:** Basic statistics of goodness-of-fit indicators for the proposed model in each of the simulations.

Statistic	Overall Success Rate	Learning Trials	Learning Time (minutes)	Time per Agent (minutes)	Success Rate after Learning
Mean	99.2	8744.4	56.2	26	99.7
Std Dev ^1^	0.6	5272.8	87.3	34.8	0.3
Minimum	98.1	500	1	1	99.1
Maximum	99.9	16,200	239	81.5	100

**^1^** Standard Deviation.

**Table 7:** Comparison of overall results of state-of-the-art approaches with the proposed model.

Simulation	Learned (%)	Overall Success Rate	Learning Trials	Learning Time (minutes)	Time per Agent (minutes)	Success Rate after Learning
Proposed model	100 (9/9)	99.2	8744.4	56.2	26	99.7
MC ^1^	88.9 (8/9)	93.6	12,546.8	63.6	29.5	98.5
TD ^2^	100 (9/9)	97.5	9546.3	97.2	45	99.2
QL ^3^	100 (9/9)	99.1	7934.6	70	32.3	99.4

**^1^** Monte Carlo; ^2^ Temporal Difference; ^3^ Q-Learning.
